# Spatio-Temporal Brain Mapping of Motion-Onset VEPs Combined with fMRI and Retinotopic Maps

**DOI:** 10.1371/journal.pone.0035771

**Published:** 2012-04-25

**Authors:** Sabrina Pitzalis, Francesca Strappini, Marco De Gasperis, Alessandro Bultrini, Francesco Di Russo

**Affiliations:** 1 Department of Education Sciences for Motor Activity and Sport, University of Rome “Foro Italico”, Rome, Italy; 2 Neuropsychology Center, Santa Lucia Foundation, IRCCS, Rome, Italy; The University of Sydney, United States of America

## Abstract

Neuroimaging studies have identified several motion-sensitive visual areas in the human brain, but the time course of their activation cannot be measured with these techniques. In the present study, we combined electrophysiological and neuroimaging methods (including retinotopic brain mapping) to determine the spatio-temporal profile of motion-onset visual evoked potentials for slow and fast motion stimuli and to localize its neural generators. We found that cortical activity initiates in the primary visual area (V1) for slow stimuli, peaking 100 ms after the onset of motion. Subsequently, activity in the mid-temporal motion-sensitive areas, MT+, peaked at 120 ms, followed by peaks in activity in the more dorsal area, V3A, at 160 ms and the lateral occipital complex at 180 ms. Approximately 250 ms after stimulus onset, activity fast motion stimuli was predominant in area V6 along the parieto-occipital sulcus. Finally, at 350 ms (100 ms after the motion offset) brain activity was visible again in area V1. For fast motion stimuli, the spatio-temporal brain pattern was similar, except that the first activity was detected at 70 ms in area MT+. Comparing functional magnetic resonance data for slow vs. fast motion, we found signs of slow-fast motion stimulus topography along the posterior brain in at least three cortical regions (MT+, V3A and LOR).

## Introduction

Visual-evoked potentials (VEPs), have been used extensively to study motion processing and integrity of the visual system. As typically reported, motion-onset VEPs have three main components called P1, N2 and P2 (reviewed in [1]). This spatiotemporal structure appears simpler when compared to other VEP stimulation modalities, such as pattern-onset, which are described by at least six components. The reason for this apparent simplicity may be due to the size and position of the stimulus in the visual field. As reported for pattern-onset and pattern-reversal VEPs also in combination with fMRI (e.g. [2], [3], [4]), small, extra-foveal stimuli encompassing less than one visual quadrant better separate VEP components.

Source analysis studies on motion VEP components concur that the origin of the N2 component is the motion-sensitive MT+ area, which includes area V5 (e.g. [5]) and may include contributions from V3/V3A or other nearby areas [6]. However, conflicting results have been obtained regarding the P1 component. Some studies have localized the P1 component in striate and extrastriate visual areas (e.g. [7]), while other studies have shown its localization in motion-specific areas (e.g. [8]). This lack of agreement among previous studies may be due to methodological differences.

The main purpose of the present study was to determine the spatio-temporal profile of motion-onset VEP and to localize its neural generators. For this purpose, we used the combined VEP/fMRI technique and stimulation paradigm (i.e., size and position of the visual stimulus) that was developed and utilized by our group in many previous studies [3], [4], [9], [10], [11]. Briefly, we used a dense electrode array and focal motion stimulation within each of the visual quadrants. Then, cortical sources were identified using dipole modeling based on a realistic head model, taking into account the loci of cortical activation revealed by fMRI in response to the same stimuli. These sources were also localized on flat maps with respect to visual cortical areas (including the recently defined area V6) identified in each individual subjects by an 0independent fMRI retinotopic mapping experiment (e.g. [12]). In addition, the classic lateral motion-sensitive cortical area MT+ was individually mapped using a dedicated functional localizer [13] to compare its functional response profile to that of the medial motion area V6 (e.g. [12], [14], [15]).

Furthermore, we addressed an important question concerning the timing of MT+ activity. As mentioned above, the majority of VEP studies concurs that the latency of the peak activity in area MT+ (represented by the N2 component) is 150–200 ms after the onset of the visual motion. This timing is surprisingly long considering that the earliest signals reach V1 with latencies of approximately 40 ms [16]. However, some electrophysiological studies have found earlier activity in MT+, ranging from 35 to 120 ms, which may bypass area V1 [7], [17], [18]. Also, neurophysiological studies on monkey have found short MT latencies to fast stimuli (e.g. [19]). These data should be taken into consideration because the V5 region in non-human primates has been shown to have anatomical connections not only from areas V1, V2, V3, V4 and V6 [20] but also directly from subcortical structures that bypass area V1, such as the lateral geniculate [21] and pulvinar [22] nuclei in the thalamus and the superior colliculus [23]. In humans, the existence and role of these direct and fast sub-cortical connections to MT+ are still unclear. However, a few studies on patients with V1 lesions have provided some evidence for the existence of such connections in humans [24]. As mentioned previously, three electrophysiological studies on healthy subjects [7], [17], [18] reported concordant evidence for early parallel inputs into MT+ that bypass area V1. However, there are discrepancies among them with respect to the onset and the peak latency of MT+ activity. Results also differ depending on whether slow or fast motion stimulation was used. Ffytche and coll. [17] reported that V5 activity began at 35 ms and peaked at 50 ms for fast moving stimuli (22°/s), while V5 activity for slow stimuli (<6°/s) began at 85 ms and peaked around 105 ms. Furthermore, Buchner and coll. [18] reported an MT+-related VEP component with an onset before 30 ms and a peak around 45 ms, but instead of motion stimuli, they used pattern-reversal checkerboard stimulation with a temporal frequency of approximately 1 Hz (1°/s). More recently, Schoenfeld and coll. [7] reported that MT+ activity began at 120 ms at and peaked at 160 ms for slow stimuli moving at 4°/s (for similar results see [25]). Therefore, a second purpose of the present study was to clarify these contradictory results concerning the onset and peak latency of MT+ activity. To address this aim, we measured the MT+ activation timing by combining VEP and fMRI data for both slow and fast-moving stimuli.

## Methods

### Subjects

Twenty-six paid volunteer subjects (mean age 23.4, range 20–32 years, 12 females) participate in the main VEPs experiment. A subset of thirteen subjects (mean age 24.6, range 21–32 years, 6 females) also received structural MRI and fMRI scanning. All subjects were right-handed and had normal or corrected-to-normal vision. Before scanning, subjects were allowed, if they desired, to consume caffeinated beverages to better maintain alertness during the scan session. Each subject participated in up to five scanning sessions.

#### Ethics statement

All participants gave written informed consent prior to both electrophysiological and neuroimaging measures, and all procedures were approved by the independent ethic committee of the IRCCS Santa Lucia Foundation of Rome.

### VEPs experiment

#### Stimuli

Stimuli consisted of a symmetrical circular Gabor pattern modulated vertically in black and white, with a visual angle of 3° in diameter (pattern cutoff), a spatial frequency of 2.4 cycles per degree and a maximum Michelson contrast of 50%. The stimulus drifted downward for 250 ms, giving the observers a clear perception of motion. Two motion speeds were used: 3°/s (slow motion) and 25°/s (fast motion). These speeds matched those used in the experiment of Ffytche and coll. [17] and were chosen because they are within the speed ranges that are processed by mostly slow or exclusively fast motion-processing channels, respectively [26].

Stimuli were presented one at a time in random order to the four quadrants of the visual field at a fast rate (stimulus onset asynchrony varying between 650 and 1000 ms). Stimulus positions were centered along an arc that was equidistant (4°) from a central fixation point and located at polar angles of 25° above and 45° below the horizontal meridian. These asymmetrical positions were chosen so that the upper and lower stimulus fields would stimulate approximately opposite zones of the lower and upper banks of the calcarine fissure, respectively, based on findings that the horizontal meridian is actually represented on the lower bank rather than at the lateral recess of the Calcarine fissure [27]. The background luminance (22 cd/m^2^) was equiluminant to the mean luminance of the pattern. Visual stimulation was displayed on a 21″ CRT monitor at a refresh rate of 144 Hz using the Presentation software (Neurobehavioral Systems, Inc. Albany, CA USA).

#### Procedure

During the electroencephalogram (EEG) recordings, subjects were comfortably seated in a dimly lit, sound-attenuated and electrically shielded room while stimuli were presented in binocular vision on a video monitor at a viewing distance of 114 cm. Subjects were trained to maintain stable fixation on a central cross (0.2°) throughout stimulus presentation and, just to keep them alert, they had to press a button with the right index finger as soon as they detected a rare and low contrast flicker of the fixation cross (150 ms duration, 2–5 s SOA). Data from these catch-trials was not analyzed. Each run lasted approximately 3 min followed by 30–60 s rest periods, with longer breaks interspersed throughout the runs. A total of 14 runs were conducted to deliver at least 525 motion stimuli to each quadrant and at each speed. Motion speeds were randomized between runs. The subjects received feedback on both their behavioral performance and their ability to maintain fixation, as monitored by electrooculograms.

#### Electrophysiological recording and data analyses

The EEG was acquired using a BrainVision™ system (BrainProducts, GmbH, Munich, Germany), with 64 electrodes placed according to the 10-10 standard montage system. All scalp channels were initially referenced to the left mastoid (M1). Horizontal eye movements were monitored with bipolar recordings from electrodes at the left and right outer canthi. Blinks and vertical eye movements were recorded with an electrode below the left eye, which was referenced to site Fp1. The EEG was digitized at 250 Hz with an amplifier and band-pass filtered between 0.1 and 100 Hz, including a 50 Hz notch filter, and data were stored for off-line averaging. Computerized artifact rejection was performed prior to signal averaging to discard epochs in which deviations in eye position, blinks or amplifier blocking occurred. We did not find differences between eyes movements recruited by the slow and fast speeds. On average, 8% of the trials were rejected for violating artifact criteria. Differences in eye movement between the slow (7.92%) and fast (8.13%) conditions were not appreciable (t_25_>1 ns). Time-locked VEP were averaged separately according to stimulus position (upper left, upper right, lower left and lower right) and speed (slow and fast). The EEG was segmented into 1100 ms epochs that began 100 ms prior the motion (voltage baseline). To reduce high-frequency noise, the averaged VEPs were low-pass filtered at 35 Hz. Data were re-referenced to averaged mastoids. VEP latency and amplitude components were measured as peak voltage deflections within specified time intervals (see results); these measures were taken at the electrode sites where the components were maximal in amplitude.

One-way ANOVAs were used to evaluate the effects of speed on each of the components, comparing slow vs. fast motion conditions for measures of peak amplitude and latency in the four quadrants. The confidence level, α, was set to 0.05 after Greenhouse-Geisser correction.

#### Modeling of VEP sources

Topographical mapping of scalp voltage and estimation of the dipolar sources of the VEP components in the grand-average waveforms were carried out using Brain Electrical Source Analysis (BESA 2000 v.5.1.8; Megis Software GmbH, Gräfelfing, Germany). The algorithm implemented in BESA estimates the location and orientation of multiple equivalent dipolar sources by calculating the scalp distribution obtained for a given dipole model (forward solution) and comparing it to the actual VEP distribution. Interactive changes in the location and orientation of the dipole sources lead to minimization of the residual variance (RV) between the model and the observed spatio-temporal VEP distribution. In the current study, this analysis used a realistic approximation of the head, with the radius obtained from the average of the group of subjects (82 mm). This realistic head model uses finite elements derived from an average of 24 individual MRIs and consists of three compartments: brain (including the cerebral spinal fluid), skull and scalp. A spatial digitizer recorded the three-dimensional coordinates of each electrode and three fiducial landmarks (the left and right preauricular points and the nasion). A computer algorithm was used to calculate the best-fit sphere that encompassed the array of electrode sites and determine their spherical coordinates. The mean spherical coordinates for each site averaged across all subjects were used for the topographic mapping and source localization procedures. In addition, individual spherical coordinates were related to the corresponding digitized fiducial landmarks and to landmarks identified on the standard finite element model of BESA 2000. The possibility of interacting dipoles was reduced by selecting solutions with relatively low dipole moments with the aid of an “energy” constraint (weighted 20% in the compound cost function as opposed to 80% for the RV). The optimal set of parameters was found in an iterative manner by searching for a minimum in the compound cost function. In addition to the RV, the quality of the model was evaluated by applying residual orthogonality tests (ROTs; e.g. [28]).

A mixed (fitting/seeding) strategy was used to model the dipolar sources of the motion VEPs. Single sources were first fit over specific latency ranges to correspond with the distinctive components in the waveform. These sources were then constrained (seeded) to the closest fMRI activation and fitted again in orientation only. To minimize cross-talk and interactions between sources, modeling followed a sequential approach according to which dipoles accounting for the earlier portions of the waveform were left in place as additional dipoles were added. Thus, the number of dipoles chosen for these models corresponded to the major topographical features of the VEP waveforms.

### fMRI Experiments

In the fMRI experiments, we used two-condition stimuli and a block sequence paradigm (eight 16 s ON, 16 s OFF epochs) for both the main motion-onset experiment and to map motion area MT+. Then, we used periodic stimuli and a phase-encoded paradigm to map retinotopy. Subjects performed three different fMRI protocols as described below:

#### Motion-onset experiment

In this fMRI experiment, the motion-onset stimulation and task were identical to those used in the VEP experiment, except for the number and duration of the runs. We tested two motion speeds (slow and fast) and only the two quadrants of the right hemifield to reduce the amount of time with the subjects lying inside the scanner for a total of four experimental conditions (upper-slow, lower-slow, upper-fast and lower-fast), each tested in separate sessions. In this procedure, 16 s of stimulation (motion-onset) alternated with 16 s of no stimulation (pattern present but stationary) for eight cycles. This sequence was repeated four times for each condition. Hence, the fMRI experiment consisted of sixteen runs of 4 min each (four runs for each condition). Prior to scanning, each subject was trained on the task (trained to maintain stable fixation and detect the flicker of the fixation cross) outside the scanner until the participant was completely familiar with the task. As with the VEP experiment, subjects were also briefly trained inside the scanner with a preliminary warm-up section.

#### MT+ mapping

Two additional scans were acquired to localize the motion-sensitive region, MT+. Stimuli produced by an X11/OpenGL program (original GL code by A. Dale, ported and extended by M. Sereno) consisted of concentric, thin, light gray rings (0.2 cycles/deg, duty cycle = 0.2) on a slightly darker-gray background, either moving (7°/s ON period) or stationary (OFF period). During the ON block, the concentric rings periodically contracted and expanded (1 s, 1 s) to avoid generating motion aftereffects during the OFF block. The average luminance of the stimulus was 61 cd/m^2^. The stimulus luminance contrast was low (∼1.5%) to better isolate MT+. It is now generally acknowledged that the relatively large motion-sensitive region found using this localizer and originally labeled V5 (or MT) in humans [13] is probably a complex of several areas (e.g. [14]). For this reason, here we referred to it as the “MT complex” or “MT+.”

#### Retinotopic mapping

We mapped polar angle (measured from the contralateral horizontal meridian around the center of gaze) and eccentricity (distance from the center of gaze) using phase-encoded stimuli, as described elsewhere (e.g. [12], [14], [29]). High contrast light and dark colored checks counterphase flickered in either a ray- or a ring-shaped configuration (polar angle and eccentricity, respectively). Stimuli moved slowly and continuously, and checks reversed between bright and dark at a rate of 8 Hz. The average luminance of the stimuli was 105 cd/m^2^. Each subject was presented with periodic stimuli (64 s/cycle, 8 cycles/scan), varying in eccentricity or polar angle, in at least two pairs of scans. This visual mapping study allowed us to define for each subject the borders of the classic retinotopically organized visual areas (V1, V2, V3, V3A, V7, VP, V4v, V4/V8) as well as the border of the newly defined visual area V6 [12].

#### Experimental set-up

Stimuli were generated by a control computer located outside the MR room, which ran in-house generated software implemented in MATLAB (The MathWorks Inc., Natick, MA, USA) using Cogent 2000 (developed at FIL and ICN, UCL, London, UK) and Cogent Graphics (developed by J. Romaya at the LON, Wellcome Dept. of Imaging Neuroscience, UCL, London, UK). Visual stimuli were projected using an LCD video projector (100 Hz refresh rate with anti-aliasing system) with a customized lens to a back projection screen mounted behind the MR tube and visible through a mirror placed inside the head coil. Stimulus luminance was calibrated to match that of the CRT monitor used in the EEG experiment. While for the motion-onset experiment we used a standard set-up (23°×12°), for both retinotopic and motion mapping we used a wide-field stimulation (up to 82° in total visual extent) similar to that described by [12]. In all experiments, fixation distance and head alignment were held constant by a chin rest mounted inside the head coil. Subjects' heads were stabilized with foam padding to minimize movement during the scans. In the motion-onset experiment, in which the subject's response was required, manual responses were collected using a magnet-compatible response pad connected to the control computer via optic fibers. Retinotopic and MT+ mapping experiment used passive viewing and continuous central fixation throughout the period of scan acquisition.

#### Imaging parameters

The MR examinations were conducted at the Santa Lucia Foundation (Rome, Italy) on a 3T MR scanner (Siemens Allegra, Siemens Medical Systems, Erlangen). Single shot echo-planar imaging (EPI) images were collected using a standard transmit-receive birdcage head coil. 30 coronal slices were 2.5 mm thick (with a 0 mm gap, interleaved excitation order), with an in-plane resolution of 3×3 mm, oriented approximately perpendicular to the calcarine fissure. Each scan took either 256 s (two-condition experiments) or 512 s (retinotopy), with 128 or 256 single-shot EPI images per slice, respectively (TR = 2000 ms, TE = 30 ms, TA = 66.6, flip angle = 70°, 64×64 matrix, bandwidth = 2298 Hz/pixel; FOV = 192). The first 8 s of each acquisition were discarded from data analysis to achieve a steady state. A total of 286 scans were carried out on 13 subjects (208 scans for the motion-onset experiment, 26 scans to map MT+, 52 scans to map retinotopy).

The cortical surface of each subject was reconstructed from a pair of structural scans (T1-weighted MPRAGE, 176 contiguous sagittal slices, 1×1×1 mm; TR = 2.00 s, TE = 4.38 ms, flip angle = 8°, matrix 256×256, bandwidth = 1130 Hz/pixel) taken in a separate session. The last scan of each functional session was an alignment scan (also MPRAGE, 1×1×1 mm) acquired in the plane of the functional scans. The alignment scan was used to establish an initial registration of the functional data with the surface.

#### Data analyses

Anatomical and functional individual data were analyzed using FreeSurfer [30], [31]. For the surface reconstruction, the two high-resolution structural images obtained from each subject were manually registered and averaged. After reconstructing each hemisphere, the inflated occipital lobe was completely flattened after first cutting it off posterior to the Sylvian fissure and making an additional cut along the calcarine fissure. Stereotaxic coordinates were calculated with an automatic nonlinear stereotaxic normalization procedure [32] using the SPM99 software platform (Wellcome Department of Cognitive Neurology, London, UK) implemented in MATLAB (The MathWorks Inc., Natick, MA, USA). The template image was based on average data provided by the Montreal Neurological Institute (MNI).

Functional individual data from two-condition experiments (i.e., Motion-onset and MT+ mapping) and phase-encoded retinotopy were analyzed based on standard procedures described in many previous studies (e.g. [14]). Briefly, P values were estimated on a voxel-by-voxel basis by constructing an F ratio between “signal” (response amplitude at stimulus frequency) and “noise” (amplitude at other frequencies excluding second and third harmonics) with degrees of freedom equal to the number of time points. The phase of the signal at the stimulus frequency was used to map retinotopic coordinates (polar angle or eccentricity). In standard block-design analysis, pseudocolor scales are usually used to represent the amplitude of the response (after masking the data with a significance threshold). The boundaries of retinotopic cortical areas were defined on the cortical surface for each individual subject on the basis of phase-encoded retinotopy and subsequent calculation of visual field sign, which provides an objective means of drawing borders between areas based on the angle between the gradients in the polar angle and eccentricity with respect to cortical position [29]. Additional affine transformations that included a small amount of shear were then applied to the functional scans for each subject using blink comparison with the structural images to achieve an exact overlay of the functional data onto each cortical surface.

Group data from the motion-onset experiment were analyzed by SPM8. Functional images from each participant were co-aligned with the high-resolution anatomical scan (MPRAGE) taken during the same session. Images were motion-corrected, transformed into MNI space using a nonlinear stereotaxic normalization procedure [32] and smoothed with a three-dimensional Gaussian filter. A standard group analysis was performed according to a general linear model, modeling “ON” blocks as box-car functions convolved with a canonical hemodynamic response function. Significance was judged by cluster size at the voxel level. Correction for multiple comparisons was performed using distribution approximations from the theory of Gaussian fields at the cluster level (p≤0.001 corrected) after forming clusters of adjacent voxels with an uncorrected threshold of p≤0.001.

Localization and visualization of individual activations by SPM were achieved using BrainShow (code by G. Galati), an in-house generated software for visualization of fMRI data. This software is implemented in Matlab (The MathWorks Inc.) and allows superimposition of SPM group maps (in MNI space, see above) on the reconstruction of the cortical surface of the average brain provided in the Population-Average, Landmark- and Surface-based (PALS) atlas and generated using SureFit and Caret software [33]. BrainShow has been used in previous studies from our and other groups (e.g. [14], [34], [35], [36], [37]) and is freely available on request for academic usage (E-mail: gaspare.galati@uniroma1.it).

## Results

### VEP waveforms and topography

#### Slow motion

**Figure 1:**
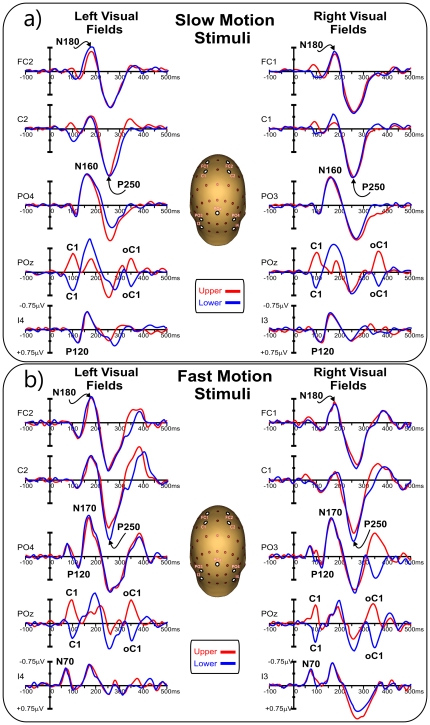
Grand averaged waveforms of the VEPs in each of the four quadrants for relevant sensor which location is indicated on the head representation. a) Response to slow motion stimuli. b) Response to fast motion stimuli.

**Figure 2:**
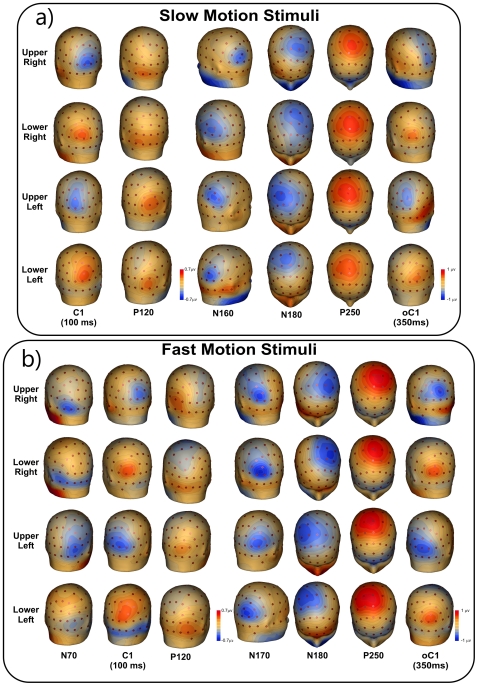
Spline-interpolated 3D voltage maps of the VEP components found in the grand averaged waveforms in the four quadrants. a) Slow motion stimuli. b) for fast motion stimuli.

The VEP waveforms elicited at relevant electrode sites by stimuli in each of the four quadrants are shown in Figure 1a, and topographical features of the major components in the scalp distribution maps are shown in Figure 2a. The earliest component (labeled C1) had an onset of approximately 60 ms and a peak latency of approximately 100 ms. In addition, the earliest component was inverted in polarity for upper vs. lower field stimuli; for upper-field stimulation, the C1 component was negative and most prominent at occipito-parietal sites slightly ipsilateral to the midline, while for lower-field this component reversed in polarity and was largest at occipito-parietal sites slightly contralateral to the midline. Partially overlapping in time with the C1 component, a positive deflection (P120) was elicited over contralateral ventral occipito-temporal sites with a peak latency of 110–120 ms. In the N2 interval between 140 and 200 ms, two temporally overlapping negative waves were elicited concurrently; an initial posterior negative peak (N160) was prominent at contralateral occipito-parietal sites, and a second negative deflection (N180) was prominent at anterior sites and had a slightly contralateral distribution over fronto-central sites. In the P2 interval between 200 and 300 ms, a large positivity (P250) dominated the waveforms. This component peaked on medial (slightly contralateral) central sites with a radial distribution. These four components (P120, N160, N180 and P250) did not invert in polarity for upper vs. lower field stimuli. Finally, activity with similar scalp distribution and polarity inversion of the C1 component peaked at 350 ms (100 ms after the stimulus offset); it is labeled oC1 in the figure 1 (offset C1).

#### Fast motion

The VEP waveforms elicited at relevant electrode sites by stimuli in each of the four quadrants are shown in Figure 1b, and topographical scalp distribution maps are shown in Figure 2b. In contrast to slow motion stimuli, the first component had an onset of approximately 40 ms and peaked at approximately 70 ms. This component, called N70, remained negative for all four quadrants and was distributed over contralateral ventral occipito-temporal sites. Additionally, the N2 complex peaked at approximately 170 ms, 10 ms later than obtained from the slow motion stimuli. However, the N160 component for the slow motion stimuli had a very similar topography to the N170 component for fast stimulation. Other than these two differences, all of the other components (C1, P120, N180, P250 and oC1) showed similar spatiotemporal features than those elicited for slow motion stimuli. Statistical comparison between conditions showed that the N160/170 and P250 components were larger for fast motion stimuli (p<0.05). Other comparisons were not significant.

### Group fMRI activations

**Figure 3:**
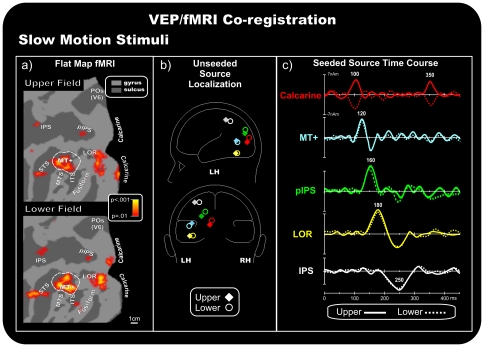
Coregistration of the VEP/fMRI responses to slow motion stimuli. a) Group-averaged contralateral fMRI activations superimposed on the flattened hemisphere (occipital lobe) of the PALS template. The pseudocolor scale indicates the statistical significance of the fMRI activations. Major sulci (dark gray) are labeled as follows: parieto-occipital sulcus (POS), intraparietal sulcus (IPS), posterior intraparietal sulcus (pIPS), superior temporal sulcus (STS), middle temporal sulcus (MTS), inferior temporal sulcus (ITS), lateral occipital region (LOR), fusiform gyrus (fusiform) and calcarine fissure (Calcarine). The dashed outline surrounding area MT+ represents the group-averaged location of the motion-sensitive cortex based on separate localizer scans. b) Schematic representation of the source locations in the unseeded dipole model. c) Source waveforms of the dipoles seeded to the fMRI activations.

**Figure 4:**
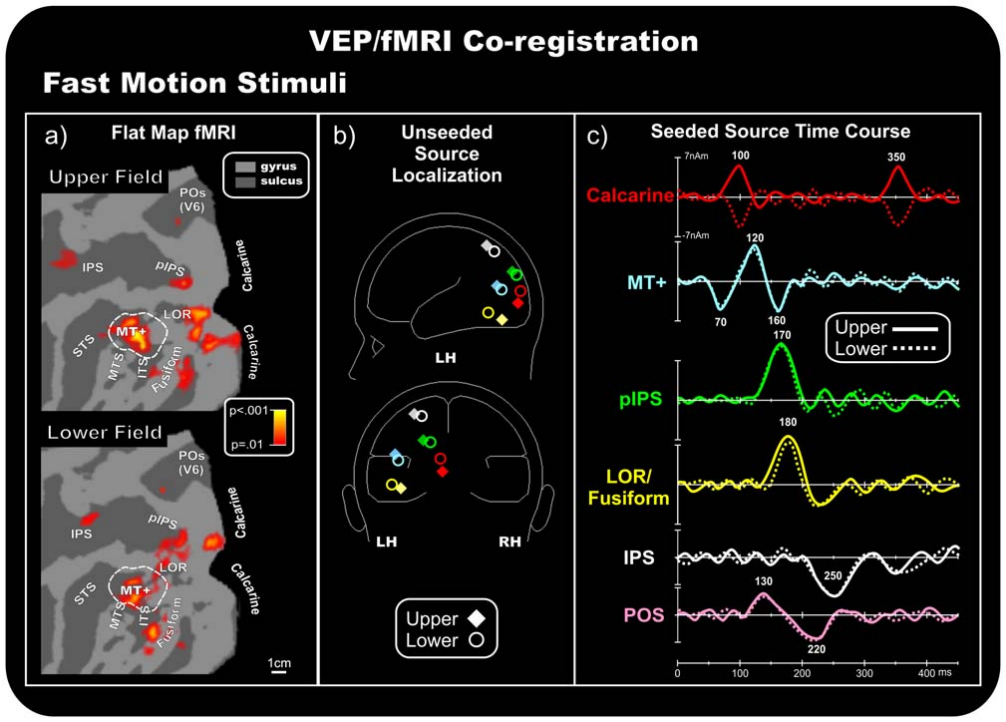
Coregistration of the VEP/fMRI responses to fast motion stimuli. For other detail, see the caption for Figure 3.

**Figure 5:**
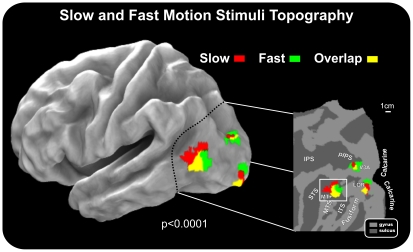
Group fMRI activations for slow and fast motion stimuli rendered on the semi-inflated cortical surface reconstruction of the left hemisphere of the average brain (left section). Results are also shown in a close-up view of the posterior part of the brain rendered on a flat map. Results from upper and lower hemifields are collapsed together. Activations for slow and fast motion conditions are plotted in different colors to represent their topographic specificity. Labels and insets are the same as those in Figure 3.

In the group-averaged data for both speeds of motion stimuli, fMRI activations were observed in multiple visual cortical areas of the contralateral hemisphere (Figures 3a and 4a), including the calcarine fissure, the MT+ complex in the temporal cortex (located in between the inferior and middle temporal sulci, ITS and MTS, respectively), the inferior occipital cortex (the fusiform gyrus and the lateral occipital regions (LOR), the middle occipital gyrus, the posterior intraparietal sulcus (pIPS, which is considered the anatomical landmark of the dorsal visual area V3A, as described in the original paper by Tootell and coll. [38] and regions more anterior in the intraparietal sulcus (IPS). For the slow motion conditions, activations were also found in the superior temporal sulcus (STS), while small activations were detected more dorsally within the parietal occipital sulcus (POS) for the fast motion condition. Figure 5 shows slow and fast motion activations (for both upper and lower hemifields) rendered together on the anatomical template (PALS). The results support a spatial segregation between the two speeds. The spatial trend is similar in the pIPS and MT+, where slow and fast motion stimuli activated the antero-dorsal and postero-ventral parts of these regions, respectively. Also, a visible spatial trend was visible in the LOR, but only in the superior-inferior direction, with the slow and fast motion activating the more ventral and dorsal portions of this region.

### VEPs/fMRI coregistration

Initially, the VEP data alone were used to create a multi-source model using the BESA algorithm (unseeded model; Figures 3a and 3b). Dipoles were optimized in the time range of 40–350 ms. Separate source models were calculated for each of the four quadrants and for the two stimulation speeds. Then, following the analysis described by Di Russo and colleagues [4], [11], regions of interest were selected by clustering the fMRI spots, and resulting coordinates (Table 1) were compared to the locations of unseeded models (Table 2) to create a final model based on the closest fMRI spot, with the source orientations optimized to the new locations (seeded model). The rationale for this strategy was to use the fMRI information to solve the inverse problem of the source of VEP localization. The quality of the matches between fMRI and VEP localization can be appreciated comparing Table 1 and 2.

**Table 1:** Talairach coordinates of the significant activations in the averaged fMRI data from thirteen subjects.

Slow Motion				Fast Motion			
Upper Right	X	Y	Z	Upper Right	X	Y	Z
Calcarine cortex	−9	−87	4	Calcarine cortex	−3	−91	−3
MT+	−45	−69	12	MT+	−45	−70	9
pIPS	−36	−83	24	pIPS	−24	−80	23
LOR	−24	−90	2	LOR	−20	−93	3
IPS	−25	−55	52	IPS	−36	−50	58
STS	−54	−43	13	fusiform	−45	−67	−10

Lower Right	X	Y	Z	Lower Right	X	Y	Z
Calcarine cortex	−6	−99	8	Calcarine cortex	−6	−96	13
MT+	−45	−67	3	MT+	−45	−70	6
pIPS	12	−80	37	pIPS	−42	−67	−7
LOR	−23	−91	−3	LOR	−18	−80	0
IPS	−31	−63	52	IPS	−30	−55	46
STS	−62	−37	18	fusiform	−38	−59	−4

Coordinates are given for contralateral activations in response to stimuli in each of the four conditions (values are in mm).

**Table 2:** Talairach coordinates of the unseeded source models based on VEP data only.

Slow Motion				Fast Motion			
Upper Right	X	Y	Z	Upper Right	X	Y	Z
Calcarine cortex	−4	−85	0	Calcarine cortex	−5	−88	1
MT+	−41	−67	8	MT+	−48	−69	12
pIPS	−34	−76	20	pIPS	−24	−80	23
LOR	−28	−70	−4	LOR	−44	−75	−3
IPS	−38	−51	56	IPS	−30	−61	63

Lower Right	X	Y	Z	Lower Right	X	Y	Z
Calcarine cortex	−5	−88	10	Calcarine cortex	−8	−90	12
MT+	−38	−70	5	MT+	−46	−73	8
pIPS	27	−78	28	pIPS	−42	−67	−7
LOR	−24	−68	−5	LOR	−40	−70	−2
IPS	−31	−59	55	IPS	−25	−65	60

Coordinates are given for contralateral activity in response to stimuli in each of the four conditions (values are in mm).

#### Slow motion

Figure 3b shows the location of the calculated best-fit sources (colored diamonds and circles) of the preliminary unseeded model for stimuli in each quadrant. Figure 3c shows the source time course (dipole moment) in the fMRI seeded locations (Figure 3a) for upper and lower quadrant stimuli. The sources were fit sequentially. First, a source was fit over the C1 range (70–110 ms), and its best fit was obtained closest to the fMRI Calcarine spot (RV max = 3.8%, 3.6% after the orientation fit). The time course of this source accounted for the C1 component well, peaking at 100 ms and inverting in polarity between the upper and lower hemifields. Furthermore, this same source also accounted for the oC1 component (340–360 ms; RV max = 3.9%, 3.5% after the orientation fit), which inverted in polarity similar to the C1 component (Figure 3c). Second, a source was fit over the P120 range (100–140 ms), with a best fit closest to the motion-sensitive MT+ complex (RV max = 2.7%, 2.4% after the orientation fit). The time course of this source accounted for the P120 component well, with an initial peak at approximately 120 ms, and partially accounted for the N160 component in the 140–170 ms time range, explaining 59.4% of variance. After a third dipole was fit in this interval, we found that the estimated location was closest to the pIPS spots (RV max = 3.7%, 3.4% after the orientation fit). The time course of this source also accounted for the peak in N160 at approximately 160 ms. Then, another source was optimized in the 170–200 ms window and its best fit was found closest to the LOR activations (RV max = 3.3%, 2.9% after the orientation fit). The time course of this source accounted for the N180 component effect well, with a main peak at 180 ms. Finally, to account the P250 component, a source was fit in the 200–300 ms window, which and resulted closest to IPS activity, showing peak activity at approximately 250 ms (RV max = 3.9%, 3.8% after the orientation fit). This multi-source model accounted for more than 96.3% of the variance of the scalp voltage topography for each quadrant over the 70–350 ms time range. The preliminary unseeded models accounted for a maximum of 95.1% of the variance in the same time range. This finding, demonstrating that the seeded dipoles only accounted for slightly more variance than the unseeded dipoles, suggests that the unseeded model accurately identified the sources of the motion VEPs that corresponded well to the sites of fMRI activation (compare Table 1 and 2).

#### Fast motion

Figure 4b shows the calculated best-fit locations of the preliminary unseeded model for stimuli in each quadrant. Figure 4c shows the source time course in the fMRI seeded locations (Figure 4a). The sources were fit sequentially. First, a source was fit over the N70 range (60–80 ms), and the best fit was found closest to the fMRI MT+ location (RV max = 3.1%, 2.9% after the orientation fit). The time course of this source accounted for the N70 component well. Furthermore, this source accounted for the P120 component and peaked at approximately 160 ms, partially accounting for the N170 component (Figure 4c).

In addition, the source analysis yielded comparable results for the other components. C1 was localized within the Calcarine fissure (final RV = 3.2% in the 70–110 ms range), and this source also accounted well for the oC1 component (final RV = 3.2% in the 340–360 ms range). The N170 was localized within the pIPS spot (final RV = 3.0% in the 140–170 ms range), and the N180 was localized within the LOR region (final RV = 3.6% in the 170–200 ms range). Finally, the P250 was localized within the IPS activity (final RV = 4.9% in the 200–300 ms range). This multi-source model accounted for more than 95.1% of the variance of the scalp voltage topography for each quadrant over the 70–350 ms time range. The preliminary unseeded models accounted for a maximum of 94.8% of the variance in the same time range.

To examine the contribution of the fMRI activity in the POS region, a further dipole was added to the model, which was seeded to the relative spot (Talairach coordinates [mm]: 18, −82, 42 and 12, −80, 37 for upper and lower stimuli, respectively), and its orientation was fit to the 70–350 ms window. The resulting time course of this POS source showed two small peak activities around 130 and 220 ms (last trace of Figure 4c). The addition of the sixth source increased the quality of the models, reducing the RV to approximately 2.4% (RV = 2.5% in the 70–350 ms range).

### Single subject fMRI activations

Stimulus-evoked fMRI activations were localized for each subject with respect to the retinotopically organized visual areas, defined on the basis of their calculated field sign, and to motion area MT+ as defined by functional localizer (see Methods). The borders of retinotopically organized visual areas (V1, V2, V3, V3A, V7, V6, VP, V4v, and V4/V8) and the border of area MT+ were identified for each participant, and activations in striate and adjacent extrastriate visual areas could be distinguished despite their close proximity and individual differences in cortical anatomy.

**Figure 6:**
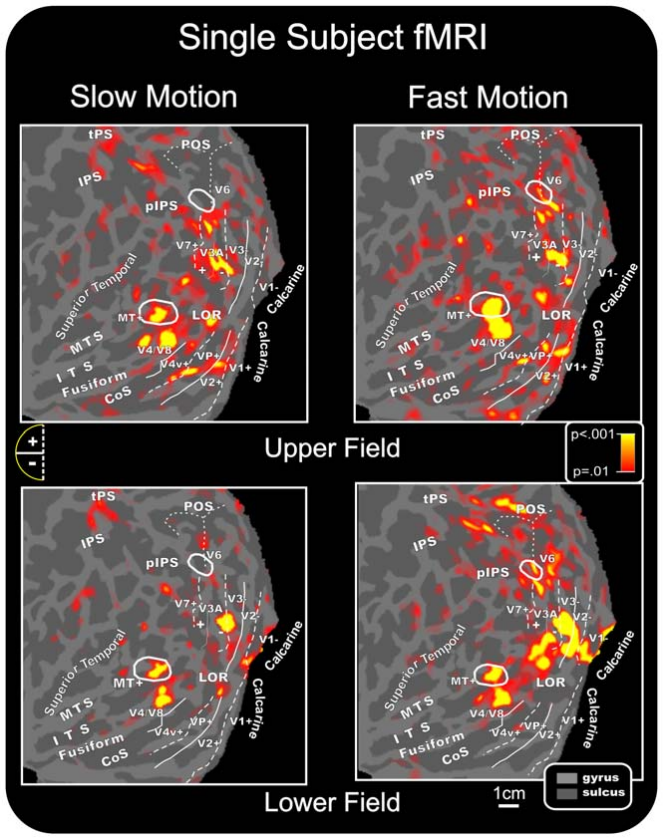
Individual fMRI activations projected onto the flattened left and right hemispheres of a representative subject. Activations in response to contralateral stimuli in each quadrant are shown in relation to the boundaries of visual areas, defined in the same subject by retinotopic mapping and consequent calculation of the visual field sign as well as by MT+ and V6 mapping. As indicated in the semicircular logos, dashed and solid lines correspond to vertical and horizontal meridians, respectively; the plus and minus symbols refer to upper and lower visual fields representations, respectively. Other labels and logos are the same as those described in Figure 5.

In individual data, slow and fast motion stimuli activated dorsal and ventral visual areas in accordance with their retinotopic representation of lower and upper portions of the visual field. As shown in Figure 6 for a typical subject, when the two motion stimuli were presented in the upper quadrants, functional responses were found in the lower banks of areas V1 and V2 as well as in areas VP, V4v and V3/V3A. This pattern of activations was consistently observed in the majority of subjects: V1+ (58%), V2+ (70%), VP (50%), V4v (50%), and V3A (80%). Stimuli in the upper quadrant also produced activations in the motion-sensitive areas MT+ (83%) and V6 (50%) as well as in the LOR (90%) and the IPS (70%). The activation observed in motion-sensitive area V6 was in accordance with its retinotopic representation of the upper visual field, which occupies the more medial part of the area [12].

When the two motion stimuli were presented in the lower quadrants, activations were consistently produced in the upper banks of V1 (90%) and V2 (80%) and in areas V3 (70%) and V3A (60%). Stimuli presented to the lower quadrants also produced activations in motion-sensitive areas MT+ (80%) and V6 (30%) as well as the LOR (88%) and the IPS (50%). The activation found in the retinotopic motion area V6 was often located in its more lateral part, superior to area V3/V3A, where the lower representation is located [12].

For both upper (70%) and lower (80%) quadrant motion stimuli, additional functional activations were observed in visual area either called V4 or V8 (and labeled in Fig. 6 as V4/V8). This ventral visual area is located just anterior to the horizontal meridian, which marks the anterior border of area V4v (e.g. [29]). Anatomically, this area is located within the collateral sulcus and extends to the fusiform gyrus. Activation of a region corresponding to this ventral visual area was also evident from the group data shown in Figures 3 and 4.

While the MT+ region was equally well activated by the two motion speeds, the activation in the motion-sensitive area V6 was observed predominantly for fast motion stimuli independently of the stimulated quadrant (being indeed present for both upper and lower visual fields). Additionally, the preference of area V6 for the fast-motion was evident both in the group data (see Figures 3a and 4a) and at the individual level, as indicated in Figure 6.

## Discussion

This study localized the main sources of the motion-onset VEPs for high and low speed stimuli by combining high-resolution EEG recordings with neuroimaging data. The spatial resolution in the present coregistration study was further increased by combining standard fMRI data with retinotopic and MT+ mapping data at the individual level. This increased resolution enabled us to localize the VEP data within each visual area and specific anatomical regions with a known functional profile, as previously demonstrated by our group in several VEP paradigms [3], [4], [9], [10], [11]. We found a complex sequence of occipito-parietal activity, including feed-forward and reentrant feedback signals highly dependent on motion speed. For slow stimuli, the processing in the cortical network started in V1 approximately 75 ms after stimulus onset, then was detected in MT+, V3A (located in the pIPS) and LOR and finally within the dorsal IPS. Upon fast motion stimulation, the processing started in MT+ approximately 40 ms after stimulus onset, then was detected in V1, and again in MT+. As for slow motion the activity was subsequently found in V3A and LOR. This path was finally completed within the dorsal intraparietal area, up to V6 in the medial parieto-occipital sulcus.

Comparing the findings from this study with previous studies on motion-onset VEP source localization, we confirmed that the early part of the N2 component is generated in MT+ [5], [7], [39]. The MT+ also generated other motion-onset VEP components, such as the P120 component for both slow and fast speeds and the N70 component, which was only present for fast stimuli. Our findings seems in line with the hypothesis that motion signals for different speeds may reach the MT+ through different pathways, either through area V1 in the case of slow stimuli or bypassing area V1 in the case of high speed stimuli [17], [24]. This feature of the motion visual system was termed *dynamic parallelism* [24]. This speculative interpretation implies that V1 does not get direct geniculate input for fast stimuli, but rather that those signals come via MT+. This is a provocative conclusion if we take the VEP dynamics literally. Moreover caution is required because this data interpretation does not relate directly to macaque physiology. Indeed, to our knowledge there are so far any animal data showing fast input to V5/MT via direct subcortical route. An alternative explanation is that both fast and slow motion signals get to V1 at roughly the same time (with fast stimulus latencies being slightly faster), but that fast signals also have a more direct route to MT+. Indeed, looking at the seeded time courses at the calcarine level (Figures 3c and 4c), fast signals peak slightly earlier than slow signals, which seems to support the conclusion that V1 gets direct geniculate inputs for both fast and slow motions (for similar interpretation see [40], but for fast motion the V1 signal might be just too weak to be seen macroscopically. On the other hand, a dedicated MT+ detour for fast motion signals should introduce additional processing delays for fast motions, but those are not seen in the time course results.

In the present study, we described VEP activity between 150 and 200 ms (the so-called motion-related N2 component) as a complex negative wave including at least three subcomponents arising from distinct areas: the aforementioned MT+, V3A and LOR, which showed peaks at 160, 160–170 and 180 ms, respectively. A similar, but simpler, N2 subdivision was also reported by Shellart and coll. [6], who based only on EEG and magnetoencephalography data, hypothesized that the N2a and N2b components originate from the extrastriate cortex, likely in or near V3/V3A and MT/V5, respectively. By combining VEP data and neuroimaging techniques, we confirmed these activities, and we showed the involvement of further activity in the LOR for the first time.

Our results showed that the C1 component was localized in area V1 with the same time course for both slow and fast stimuli. These results confirm the findings from the only previous study that reported early activity for motion stimuli; there, this early activity was defined the C1 as well [7]. It is evident from the extensive VEP literature on this topic (see introduction) that the C1 has been found in response to many visual stimulation paradigms, such as pattern-onset, pattern-reversal and motion-onset [3], [10]. Therefore the C1 seems to be a ubiquitous phenomenon related to the activity of the V1 area and it would reflect the cortical volley from the lateral geniculate nucleus.

Additionally, we identified for the first time the origin of the P2 (P250) component in the dorsal IPS with a peak at 250 ms. This result is in agreement with a previous hypothesis relating this component to a higher order visual processing level, similar to that of biologically important stimuli [1]. According to single-cell data from monkeys [41], dorsal IPS areas are involved in the integration of multimodal information for constructing a spatial representation of the external world. In monkeys, these areas serve as interfaces between the perceptual and motor systems for controlling arm and eye movements in space. In humans, many fMRI studies have shown that the IPS is composed of a mosaic of areas subserving goals similar to those described for analogous regions in the monkey (e.g. [35], [42]). The human dorsal IPS areas are involved in spatial attention and visuo-motor control (e.g. [43]), contain visuo-topic maps of contralateral space (e.g. [44]) and are involved in goal-directed stimulus and response selection [45].

Lastly, in the fast speed condition only, two responses at 130 and 220 ms were accounted for, at least in part, by a source near the POS, which corresponds to the visual area V6 [12]. Recently, we detected the electrophysiological correlates of the human area V6 [11] and showed that the neural response in this area occurred approximately 200–250 ms after stimulus presentation, which matches the findings in the current study. Furthermore, recent work by Pitzalis and coll. [14] has shown that, as in primates, the human V6 is a motion-sensitive area, which responds much more strongly to coherent flow fields than incoherent random motion. Additionally, the present results show that area V6 responds to fast speed motion stimuli and independently to the visual quadrant stimulated. It is possible that the high speed motion stimuli resembled a flickering visual stimulation, which is known to activate area V6 (e.g. [14]) and other motion areas. Indeed, motion-sensitive cells typically respond rapidly and transiently to stationary stimuli and, thus, are activated by flickering stimulations [46]. Results seem suggest a *selective* preference of human area V6 to fast speed motion. However, caution is required because the effects are small. Note indeed that the average signal in area V6 (see Fig. 4) was weak not surviving indeed at higher threshold used in Fig. 5. This was likely due to the small size of the stimuli used here which have surely penalized the response in human V6 which (like macaque V6) represents the fovea but emphasizes the visual periphery (e.g. [12]).

In macaque, preliminary observations showed the presence in area V6 of classes of cells responding to different speeds, from very low (about 1 deg/s) to very high (more than 100 deg/s; [20]. In contrast, here we did not find speed-related organization in area V6 being the area silent for the slow motion. Alternatively, It might be that, because of the strict speed selectivity of V6 cells [20], the total number of cells activated by a single speed moving stimulus in the fMRI experiments is small, likely much less than the total number of cells activated by the Flow Field stimulus, where direction and speed of movement, as well as type of movement coherence, changed every 500 ms. In other words, it is possible that to optimally activate V6 we had to use a range of velocities instead of a single speed, in the attempt to activate as many speed-sensitive neurons as possible with a single stimulus. Overall, the use of one-speed stimulus together with the small size of the stimuli used here surely penalized the area and likely explain the lack of response in area V6 for the low speed motion stimuli and the general weak average signal we observed in the area.

It should be cautioned that the use of hemodynamic imaging to substantiate the estimated locations of ERP sources, as was done in the present and previous studies [3], [4], [9], [10], [11], [47], [48], [49], [50], [51], [52] is subject to certain caveats [53], [54]. First and foremost is the assumption that the hemodynamic response obtained with fMRI or PET is driven by the same neural activity that gives rise to the ERP. With regard to visual-evoked activity, such a correspondence appears to be optimal for human medial occipital cortex (including the Calcarine fissure) and is less definite for extrastriate visual areas [55]. Moreover, it stands to reason that a more accurate source model can be achieved for the initial VEP component than for subsequent components, which receive contributions from multiple, spatially and temporally overlapping cortical generators.

### Cortical areas involved in speed perception

Neuroimaging studies have shown an extensive network of cortical areas that are responsive to moving stimuli in the human brain [13], [14], [56], [57], [58]. However, study of neural mechanisms that underlie different speed channels has generally focused on the role of V5/MT; the response of other motion-sensitive areas as a function of different speeds remains mostly unexplored. Here, we found signs of slow-fast motion stimulus topography along the posterior brain in at least three cortical regions (MT+, V3A and LOR). These regions were activated differently or in different parts depending on the speed of the motion stimuli.

The clearest and most intriguing result was observed in the motion-sensitive region MT+, which was differentially activated by stimulus velocity; the antero-dorsal part was sensitive to slow motion and its postero-ventral portion was sensitive to fast motion. The middle portion of MT+ was always activated by motion stimuli independent of speed. This segregation is congruent with the slight difference found between the VEP topographies of the fast-motion N70 component and the motion-related slow motion component. Present results are in line with previous evidence supporting a role for MT in speed perception in both monkey and human brain. In nonhuman primates, stimulus speed modulates the activity of most MT neurons (e.g. [59], [60], [61]), and lesions to this area can impair performance on speed discrimination tasks and pursuit tasks (e.g. [62], [63]). In the human brain, some functional imaging studies have implicated the MT complex in speed discrimination (e.g. [64]), but this finding is not consistent across studies (see [65] for negative evidences). Interestingly, and in line with present results, McKeefry and coll. [66] found that application of repetitive transcranic magnetic stimulation (rTMS) to area V5/MT induced deficits in speed perception and increases in speed discrimination thresholds. Signs of velocity segregation of MT+ observed here are a novel finding that could be evocative of a *speed-topic* organization of the MT+. Single-cell studies indeed show clustering of speed-tuned cells in MT, even though that occurs at a much smaller spatial scale than the present results (e.g. [61]). Of course we are well aware that speed-topic mapping implies a smoothly changing representation of speed from slow through medium to fast as one moves across the cortical surface. This has not been demonstrated here where only two speeds were used. An alternative explanation is that present results likely show different speed preferences of different areas within MT+ complex. It is now generally acknowledged that the relatively large motion-sensitive region originally labeled V5 (or MT) in humans [13] is a complex of several areas—the “MT complex” or “MT+.” This assumption is based on both the known functional subdivision of the MT+ in MT and MST (e.g. [64]) and the discovery of a mosaic of small retinotopic areas around retinotopic MT/V5 found in recent mapping papers (e.g. [14]). So the difference between the fast and slow motion related activations could reflect two discrete functional areas inside MT+ (V5/MT and MST) or inside the same MST (e.g MSTd and MSTl). These different areas might prefer different speeds but that does not imply a “map” of speed. Of course, to really understand and explain the speed-tuning results shown in this study, one would have to look at separate sub-areas that comprise the MT+ complex (e.g. [14], [64]). This is out of the scope of the present paper, where the ERP/fMRI coregistration is the main target. The limited spatial resolution of the ERP methods would not benefit from a fine spatial segregation between MT and MST provided by the fMRI methods. In summary, while speed selective activations are interesting, they are really just intriguing preliminary results. Future fMRI dedicated experiments will be necessary to reveal the presence of ‘maps’ of speed in the MT+ complex. The spatial trend observed in MT+ is similar to that seen in area V3A, where the antero-dorsal part was sensitive to slow motion and its postero-ventral portion was sensitive to fast motion. The speed organization found in area V3A is consistent with McKeefry and coll. [66] who reported that the application of rTMS to area V3A induced deficits in speed perception analogously to area MT+. The motion sensitivity in area V3A has been observed in several fMRI studies [14], [29], [58]. Much is still unknown about the exact nature of its contribution to motion perception but it seems to have speed sensitivities almost identical to MT [67]. This speaks against psychophysical data from humans suggesting two separate systems processing fast and slow speeds (e.g. [26]) whereas it is in line with more recent psychophysical models (e.g. [68]) and neurophysiological recordings in monkeys (e.g. [61]) generally showing continuous speed representation, hence hinting at a single system. Alternatively, as hypothesized for MT+, the *speed-topic* organization observed in V3A could show different speed preferences of different areas within V3A. In a previous papers [14], we showed that optic flow activated the anterior part of V3A while radial motion activated the posterior part of V3A, as previously observed also by Sereno and coll. [44]. Given these data, it is possible that V3A as originally defined (e.g. [38]) contains more than one visual area. The similar pattern of results observed in both area MT+ and V3A represents a significant finding. So far, area V5/MT has been considered the main cortical locus for the neural mechanisms that underlie the perception of stimulus speed. The results presented here demonstrate that stimulus speed is also encoded within area V3A solidifying the prominent status of V3A in the cortical network that exists for the processing of motion in the human brain [38], [69], [70]. A similar but less clear-cut spatial trend was also visible in the LOR, although only in the posterior-anterior direction. Specifically the anterior part was sensitive to slow motion and the posterior portion was sensitive to fast motion. The LOR refers to the cortex between dorsal areas V3 and MT+ [70], [71], [72]. This cortical region is located on the lateral occipital sulcus, in between dorsal and ventral visual areas, and overlaps retinotopic dorsal areas, such as the V3B [70] and V4d [71]. The LOR is also part of the kinetic occipital motion-sensitive region described by Orban's group [72]. Also, in a recent study by our group [14], a motion-selective response in the LOR for radially moving stimuli was found. So far, there have been no other previous studies on the speed sensitivity of the LOR. Like for MT+ and V3A, also in this case a possible interpretation of the slow-fast motion stimulus topography observed in the group data (see Fig. 5) can be interpreted in light of the LOR subdivision in sub-regions proposed by different authors in the last ten years (e.g. [71]: LOC and LOP, [73]: LO1 and LO2).

Taken together, these results suggest that areas MT+, V3A and LOR play an important role in a cortical network that underlies the speed processing in the human brain. Future and dedicated fMRI experiments will be needed to better investigate the functional organization of the speed processing in the human brain and the role these three cortical regions (and their subdivisions) play in the perception of speed.
